# Case report: tracheobronchial diverticulum, a potential risk for diving?

**DOI:** 10.3389/fmed.2023.1340974

**Published:** 2024-01-11

**Authors:** Long Qing, Yan Wang, Meng Zhang, Qinqin Pu, Wanwan Cai, Yaping Pan, Delin Xia

**Affiliations:** ^1^Department of Naval Diving Medicine, Naval Medical Center, Naval Medical University, Shanghai, China; ^2^Department of Respiratory Critical Care Medicine, Naval Medical Center, Naval Medical University, Shanghai, China

**Keywords:** tracheobronchial diverticulum, barotrauma, fitness to dive, diving, computed tomography, decompression

## Abstract

Tracheobronchial diverticulum (TBD) is an asymptomatic, benign cystic lesion outside the lumen of the trachea and bronchus. This is the first report case of a SCUBA (self contained underwater breathing apparatus) diver diagnosed with TBD, which is a potential risk to diving. No literature or guideline is available so far on the diving fitness for patients with congenital or acquired TBD condition. A healthy 26-year-old male professional diver has records of SCUBA diving up to a depth of 40 meters sea water. He did not have any diving-related injuries or symptoms during his career and had no history of smoking, drinking, or other special illnesses except for a COVID-19 infection. A tracheal diverticulum was found accidentally by computed tomography (CT), but its communication with the trachea was not clear initially. Therefore, high-resolution CT and electronic bronchoscopy were done to clarify the situation of the diverticulum and identify the diving risk. High-resolution CT showed a possible opening in the diverticulum, but this was not seen under electronic bronchoscopy. Although a potential opening was shown in high-resolution CT, the lack of visual bronchoscopic evidence made it likely to be a dead cavity. As there is a higher theoretical risk of barotrauma during decompression, leading to pneumomediastinum, hemorrhage, or arterial gas embolism, the current clinical consensus is that air-containing tissue should be regarded as a relative contraindication for diving. Overall, it is recommended that the diver should dive carefully and avoid ascending too rapidly.

## Introduction

The respiratory system has always been a crucial concern of divers and diving physicians (1). Once an anomaly is found, it will not only threaten the safety of the diver and affect their confidence but also disqualify a diver from diving. Therefore, physicians should look for abnormalities and make a clear judgment about the diver’s fitness to dive (2, 3). When no guideline is used to specify or regulate a special disease that may threaten diving safety, the judgment can be challenging for the reason that a diver’s hobby or career should not be abruptly ruined, but they should also not be exposed to a high risk of diving accidents.

A tracheobronchial diverticulum (TBD) is a cystic lesion that involves the tracheobronchus and protrudes outside it (4). A TBD usually has no clinical symptoms and is only found on computed tomography (CT) examination. It is a benign lesion caused by local tracheobronchial wall weakness and swelling and there is no accurate report of its incidence at present. Parts of the diverticulum may not communicate with the tracheobronchial cavity or have compartments, which are physiologically closed spaces that need to be carefully evaluated in diving medical examinations (5).

Presently, no literature or guideline governs the fitness to dive for individuals with a TBD. This is the first case report of a SCUBA diver diagnosed with a TBD, which is a potential risk to diving. In theory, minor lesions may cause local bleeding and laceration due to barotrauma, and severe lesions may cause asphyxia, pneumothorax, mediastinal emphysema, arterial air embolism, etc. This case report also introduces diving medical expert opinions on such an individual’s fitness to dive.

## Case presentation

### Medical history

The asymptomatic 26-year-old male professional diver in this case report had a history of SCUBA (self contained underwater breathing apparatus) diving for 6 years (10∼15 times per month, about 800 dives in total) with a maximum dive depth of 40 meters sea water (msw) and an average depth of 10 msw. The diver declared no rapid decompression experience, history of decompression illness, or discomfort symptoms after diving including cough, expectoration, or hemoptysis. He did not smoke or drink alcohol. There was no past surgery or special illnesses except for a COVID-19 infection 3 months earlier, the symptoms for which included a fever for a week, a sore throat for 4 days, and a cough and sputum for nearly 10 days. The chest X-ray of the previous annual physical examination was unremarkable according to the Chinese national standard “criteria of physical examination for commercial divers.”

### Investigation

**FIGURE 1:**
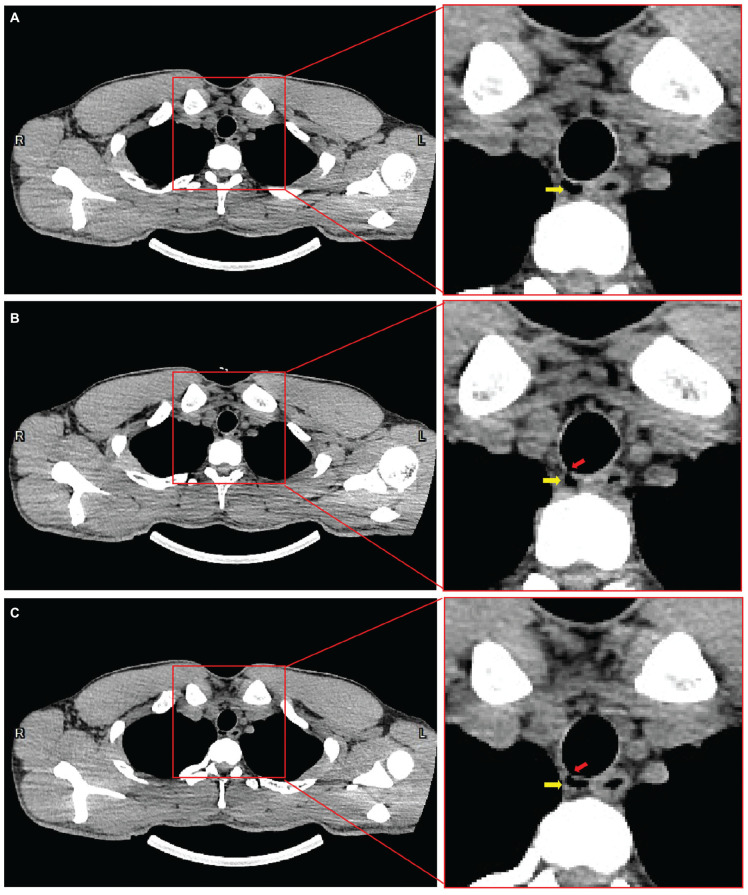
Mediastinal window of 640-slice chest computed tomography revealing tracheal diverticulum position and possible communication with trachea. The tracheal diverticulum is located on the right posterior side of the upper segment of the trachea, adjacent to the esophagus **(A)**. Possible communication with the trachea was found in two level two scanning layers, and the widths of the openings were approximately 1.8 mm **(B)** and 1.1 mm **(C)** as marked by red arrows. The diverticulum is marked by yellow arrows in **(A,B,C)**.

**FIGURE 2:**
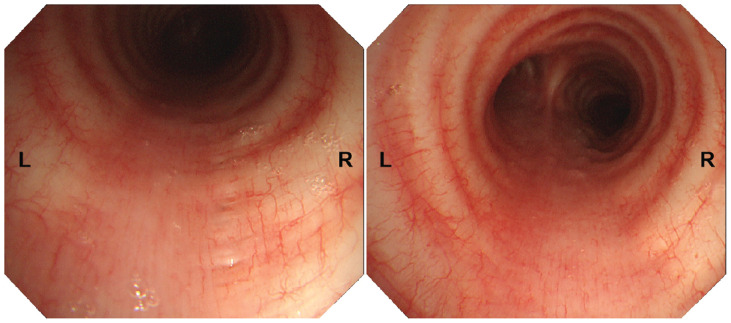
Electronic bronchoscopy revealing no prominent opening in the tracheal diverticulum.

In March 2023, a 64-slice CT of the chest was performed to evaluate the influence of COVID-19 infection on the lungs at a local hospital on the advice of a local diving physician. This was the first time the diver found the TBD, located on the right posterior side of the upper segment of the trachea. The size of the TBD was reported to be 0.3 cm × 0.6 cm × 1.2 cm. CT showed that the diverticulum may communicate with the trachea. In order to make a clear definition of the character of the TBD, a high-resolution (640-slice) CT examination was performed in our hospital. It showed a small, air-containing transparent cavity without separation, almost the same size as the last CT report, in the right posterior aspect of the trachea in the upper mediastinum, approximately 4 cm from the carina of trachea (Figure 1A). Possible communication with the trachea was found in two levels of scanning layers, and the widths of the openings were approximately 1.8 mm and 1.1 mm (Figures 1B, C). Electronic bronchoscopy was further performed after obtaining the patient’s consent. Repeated observations were made at the location where CT showed possible openings of the diverticulum, and no obvious holes or surface depressions were observed (Figure 2 and Supplementary Figures 1–4). There were no obvious abnormalities in other examinations, including a pulmonary function test, routine blood test, and serum biochemistry.

### Outcome

The diver was not prohibited from diving but was clearly informed of a risk of barotrauma and mediastinal emphysema due to complications from the lesion. The diver was advised that caution should be exercised in the future, that they should avoid diving when suffering from respiratory tract inflammation, and absolutely prevent a rapid ascent. Continuous re-examination was recommended at least once a year. Up to the time of submission of this report, he had safely dived nearly 50 times.

## Discussion

Diving is associated with substantial changes in ambient pressure (5). Normally, the gas in the body is balanced with the external pressure through the respiratory system and circulatory system. The effects of Boyle’s law result in changes in the volume of gas-containing spaces when exposed to the changed pressure underwater, which may lead to extrusion or tear of tissues and organs (6). Hence, when considering diving safety, special attention should be paid to the abnormal or closed gas cavity of the diver’s body, which will pose a threat to diving safety. A TBD is one such potentially dangerous abnormality, which is not uncommon, but there has been no report or discussion about its association with diving risks. A TBD is a benign lesion caused by local tracheobronchial wall weakness and swelling (4, 7). It is usually divided into congenital or acquired. Most of the congenital diverticula are small and have similar tracheobronchial anatomical structures, including the airway wall, smooth muscle, and columnar epithelium, in which a defect of elastic fibers of the tracheobronchus or muscle fibers is a potential factor (8). Most acquired diverticula are larger, and their lumen walls are mainly composed of tracheal epithelium rather than smooth muscle and cartilage, which are often associated with chronic obstructive pulmonary disease, and mucus glands in the trachea proliferate, causing dilation and gradually forming a diverticulum (5). A TBD is common in the posterior wall of the trachea, the gap in the cartilage ring of the trachea, or the membranous part of the trachea. The diverticulum may have a locular or cellular change and a pleated or compartmental structure, and the outer wall is usually thin and clearly delimited from the surrounding tissue. A TBD usually has no significant clinical symptoms and is usually found by CT examination due to chest diseases or health checkups (9). Diverticular infection may occur in severe or long-term recurrent upper respiratory tract infection (10).

The diverticulum could theoretically be a timebomb for the diving population. As outlined above, if there is a closed gas cavity in the body, pneumatic injury may occur due to the imbalance of internal and external pressure during decompression. Barotrauma can cause pain and bleeding in other areas, such as the sinuses, teeth, and gastrointestinal tract (11), but the occurrence of pulmonary barotrauma is more serious (12). Pulmonary barotrauma may cause pneumothorax, mediastinal emphysema, arterial air embolism, etc., and it is highly possible that it could lead to life-threatening consequences (12). For this reason, divers and physicians pay special attention to respiratory diseases, such as pulmonary bulla and emphysema (13). A TBD poses a similar risk if it ruptures as it is a part of the respiratory system. Therefore, it is necessary to determine whether the diverticulum is a closed cavity, in communication with the tracheobronchus, and whether there is internal separation. Even if there is an opening and no separation, it may still be blocked by secretions, forming a closed space when a diverticular infection occurs. In this case, the patient wanted to identify the diverticular opening to assess the risk of diving.

At present, high-resolution CT is considered to be the best way to diagnose TBD, which is characterized by irregular or circular-like low-density shadows consistent with gas density in the tracheobronchial lumen (9). It can show the location, size, number, contents, wall, and surrounding tissue of the diverticulum. Chest X-ray has limited potential in both finding and diagnosis. Chest magnetic resonance imaging (MRI) can also detect a tracheobronchial diverticulum, but because it is not as thick as high-resolution CT, the detail of the diverticulum is poor (12). However, it is better in the comparison of the diverticular wall, internal infection, and therapeutic effect. However, these radiological examinations cannot fully define the communication of the diverticulum, especially the small opening. The thin membrane at the opening may also not be visualized on high-resolution CT or even MRI. In this case, two CT examinations showed ambiguous openings. Electronic bronchoscopy is another option, which can visually observe the opening of the diverticulum. It is usually manifested as small holes or surface depressions in the tracheobronchial wall (14). However, the ability to detect abnormalities is also related to the experience of the operator and the clarity of the equipment. The bronchoscopy of the diver did not reveal any abnormalities.

For the general population, asymptomatic patients generally do not need to be treated, and those with inflammation can be treated with anti-inflammation, postural drainage, and other conservative treatments (8). Surgical treatment is rarely required, unless severe infections, abscesses, or fistula are found (15). For divers, there are no standards or guidelines to clarify the diving-related issues of a TBD. As with pulmonary bulla and emphysema, there is a theoretical risk from a TBD when diving, but there has been no adequate clinical research or epidemiological investigation (13). In view of the special conditions for the onset and extremely small incidence of diving diseases, TBD is also a theoretical risk but lacks clinical evidence. In order to ensure the safety of the diver, the first priority is to clarify the details of the diverticulum, especially the internal separation and opening into the respiratory tract and the adjacent relationship with the surrounding tissue. No intervention can be done temporarily for recreational divers. However, for professional, military, and technical divers, attention should be paid to the possible risks of a closed diverticulum and surgery may be an option if appropriate. For the diverticulum with a small opening, diving should be avoided in the presence of respiratory tract infection, and the status of the diverticulum should be reviewed regularly, especially after infection, to prevent the formation of a flap or membrane at the opening, or internal separation after inflammation. These recommendations remain theoretical in the absence of any cases of TBD resulting in diving injury. If an individual with a TBD had been SCUBA diving for many years without any injury, there may either be a tiny opening to balance pressure, which is too small to be detected, or it may be a TBD in the outer wall which is elastic enough to compress and expand without injury during pressure changes. The diver in this case has not been prohibited from diving but advised as described above including re-assessment at least once a year. From an epidemiological perspective, TBD has a certain incidence rate, and there must be many unproven asymptomatic divers, however, there have been no reports of diving injuries caused by TBD. Hence, conservative advice is not beneficial for divers.

In summary, the danger of TBD for diving remains theoretical. Countermeasures should be analyzed according to the diverticulum but also depend on a diver’s acceptance of risk and the physician’s views.

## Data availability statement

The datasets presented in this study can be found in online repositories. The names of the repository/repositories and accession number(s) can be found in this article/Supplementary material.

## Ethics statement

Written informed consent was obtained from the individual(s) for the publication of any potentially identifiable images or data included in this article. Written informed consent was obtained from the participant/patient(s) for the publication of this case report.

## Author contributions

LQ: Conceptualization, Funding acquisition, Project administration, Writing – original draft. YW: Conceptualization, Data curation, Writing – original draft, Writing – review & editing. MZ: Investigation, Formal analysis, Writing – original draft. QP: Investigation, Writing – review & editing. WC: Data curation, Writing – original draft. YP: Validation, Writing – original draft. DX: Data curation, Writing – original draft.

## Supplementary material

Supplementary Figures 1–4 Electronic bronchoscopy found no abnormalities in the trachea.
