# Supplementary figures and images for: Case report: tracheobronchial diverticulum, a potential risk for diving?
Source: Front Med (Lausanne). 2024 Jan 11;10:1340974. doi: 10.3389/fmed.2023.1340974 (PMC10808740; doi:10.3389/fmed.2023.1340974)

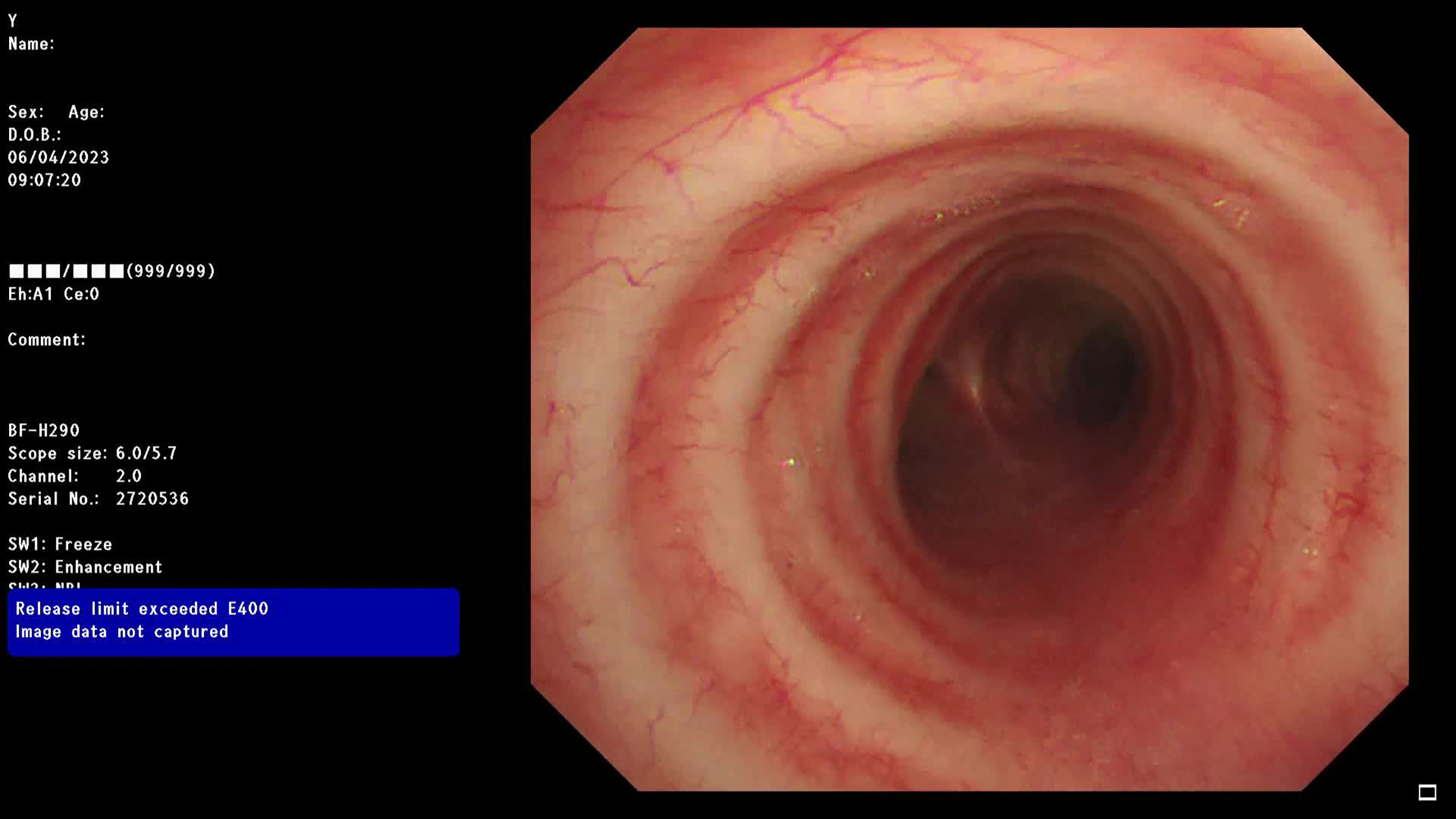

Supplement: Supplementary file 1 [file Image_1.JPEG]

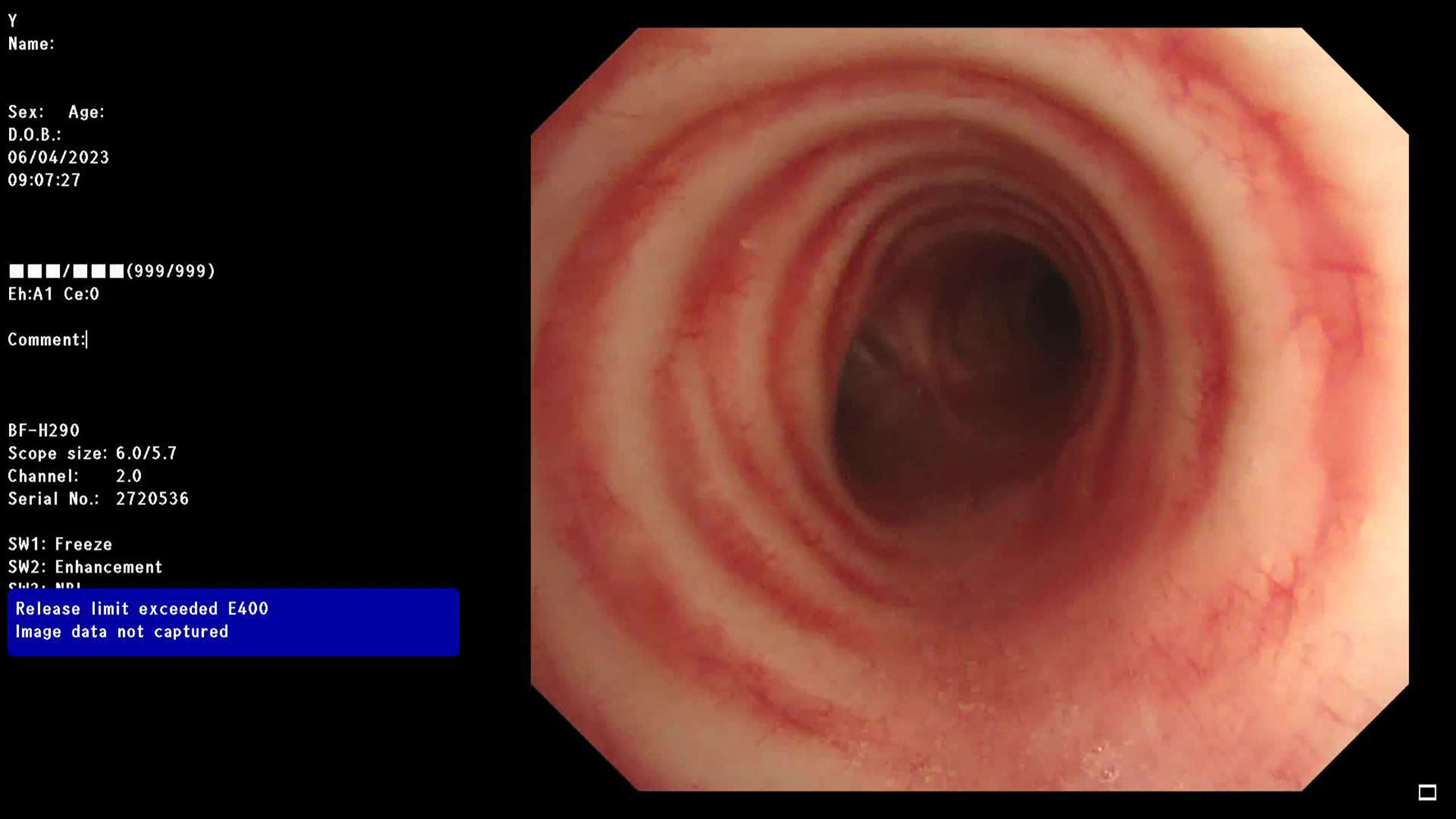

Supplement: Supplementary file 2 [file Image_2.JPEG]

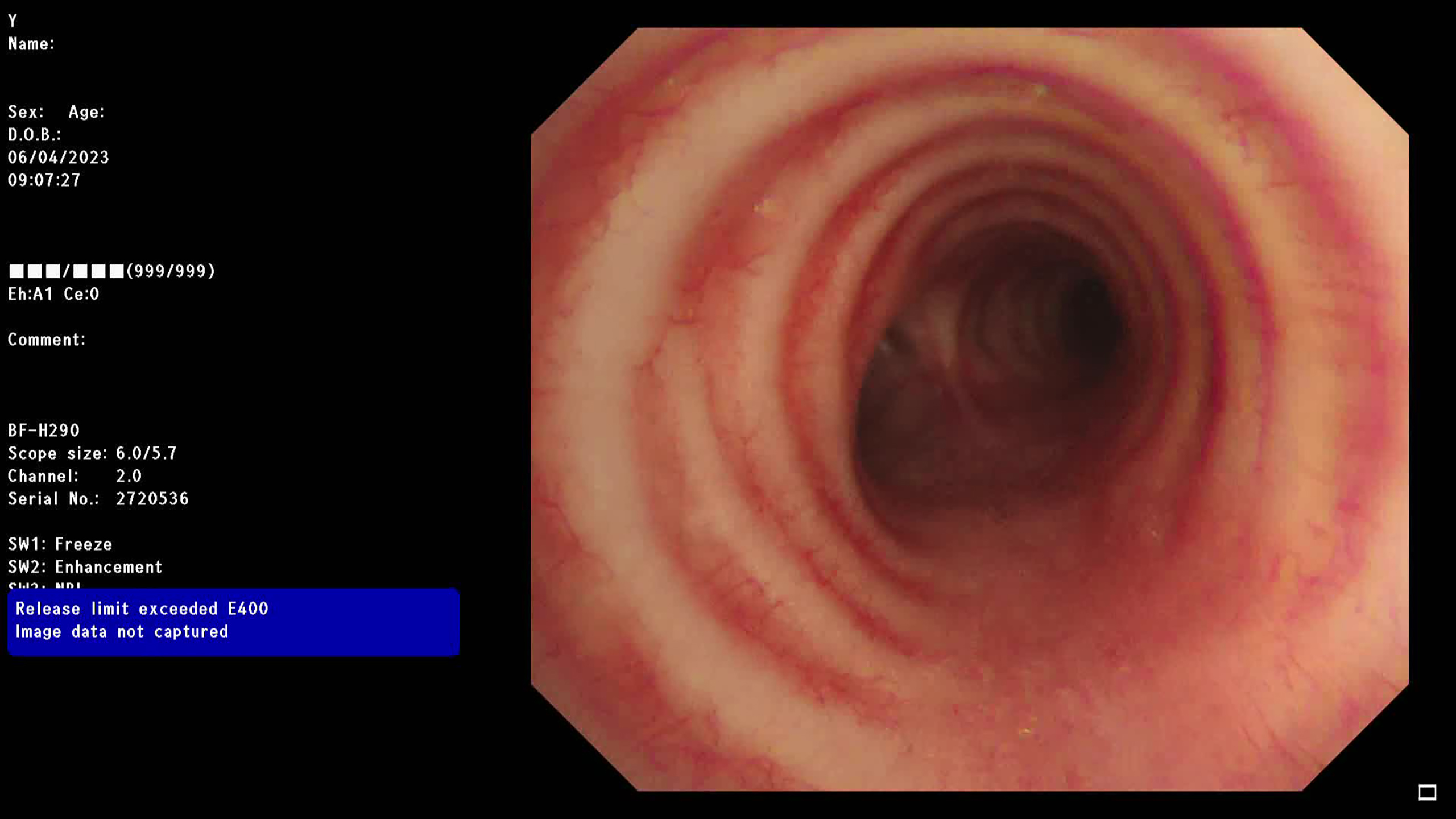

Supplement: Supplementary file 3 [file Image_3.JPEG]

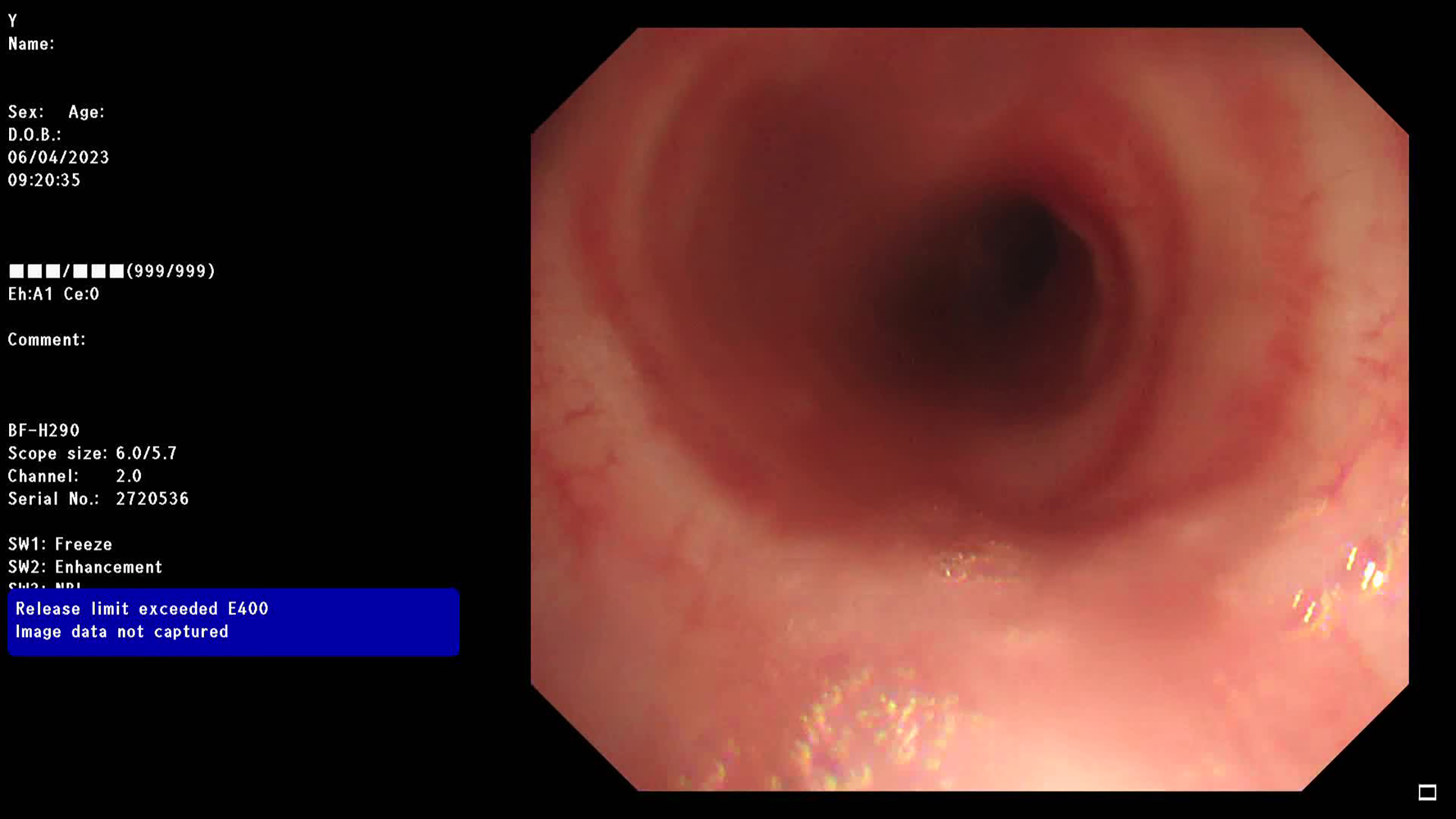

Supplement: Supplementary Figures 1–4 — Electronic bronchoscopy found no abnormalities in the trachea. [file Image_4.JPEG]
